# Prognostic value of hematogenous dissemination and biological profile of the tumor in early breast cancer patients: A prospective observational study

**DOI:** 10.1186/1471-2407-11-252

**Published:** 2011-06-16

**Authors:** Montserrat Solá, Mireia Margelí, Eva Castellá, Juan F Julian, Miquel Rull, Josep M Gubern, Antonio Mariscal, Agustí Barnadas, Manuel Fraile

**Affiliations:** 1 Nuclear Medicine Department, University Hospital Germans Trias i Pujol, Carretera del Canyet, Badalona, Spain; 2 Oncology Department, Catalan Institute of Oncology, University Hospital Germans Trias i Pujol, Carretera del Canyet, Badalona, Spain; 3 Pathology Department, University Hospital Germans Trias i Pujol, Carretera del Canyet, Badalona, Spain; 4 Surgery Department, University Hospital Germans Trias i Pujol, Carretera del Canyet, Badalona, Spain; 5 Surgery Department, Mataró Hospital, Carretera de Cirera, Mataró, Spain; 6 Radiology Department, University Hospital Germans Trias i Pujol, Carretera del Canyet, Badalona, Spain

**Keywords:** Breast cancer, Disseminated tumor cells, Bone marrow, Sentinel node, Survival

## Abstract

**Background:**

The aim of this study was to investigate the incidence and prognostic value of disseminated tumor cells in bone marrow of breast carcinoma patients with early disease, and to analyze this finding in relation to lymph node involvement, determined by sentinel lymph node (SLN) biopsy analysis, and to prognostic factors of interest.

**Methods:**

104 patients with operable (T < 3 cm) breast cancer and clinically- and sonographically-negative axillary lymph nodes were scheduled for SLN biopsy. Bone marrow aspirates were collected before the start of surgery from both iliac crests, and mononuclear cell layers were separated by density centrifugation (Lymphoprep). Slide preparations were then examined for the presence of disseminated tumor cells by immunocytochemistry with anti-cytokeratin antibodies (A45-B/B3). Lymphoscintigraphy was performed 2 hours after intratumor administration of 2 mCi (74 MBq) of 99mTc colloidal albumin. The SLN was evaluated for the presence of tumor cells by hematoxylin-eosin staining and, when negative, by immunocytochemistry using anti-cytokeratin antibody (CAM 5.2). Survival analyses and comparative analyses were performed on the results of bone marrow determinations, SLN biopsy, and known prognostic factors, including breast cancer subtypes according to the simplified classification based on ER, PR and HER2.

**Results:**

Lymph node and hematogenous dissemination occur in one-third of patients with early-stage breast cancer, although not necessarily simultaneously. In our study, disseminated tumor cells were identified in 22% of bone marrow aspirates, whereas 28% of patients had axillary lymph node involvement. Simultaneous lymph node and bone marrow involvement was found in only 5 patients (nonsignificant). In the survival study (60 months), a higher, although nonsignificant rate of disease-related events (13%) was seen in patients with disseminated tumor cells in bone marrow, and a significant association of events was documented with the known, more aggressive tumor subtypes: triple negative receptor status (21%) and positive ERBB2 status (29%).

**Conclusions:**

Tumor cell detection in bone marrow can be considered a valid prognostic parameter in patients with early disease. However, the classic prognostic factors remain highly relevant, and the newer breast cancer subtypes are also useful for this purpose.

## Background

Breast cancer is a leading cause of death in middle-aged women in developed countries. The incidence of this condition appears to show an upward trend, and it is now one of the priority issues in community health. In Spain, breast cancer is the most common malignancy in women and the leading cause of cancer deaths. The incidence rate has risen from 54.8 cases per 100,000 woman-years in 1980-1984 to 83.1 in 2000-2004 [1]. Mortality has shown a downward trend from 1992 to 2005, but it still reaches rates of 27.4 per 100,000 women [2].

Early detection of breast cancer is the basis of effective treatment. Recognized prognostic indicators, such as tumor size, grade and histological type, and steroid hormone receptor status are defining characteristics of tumor proliferation and the degree of differentiation; hence, these parameters provide indirect information on the capability of the tumor to metastasize.

The axillary lymph node status, determined by sentinel lymph node (SLN) biopsy or complete axillary node dissection, is currently the most widely accepted direct indicator of breast cancer spread. Lymph node involvement is, therefore, a prognostic indicator of the risk of systemic disease. Survival curves according to lymph node involvement are classic descriptors of the natural history of breast cancer [3].

In contrast, consolidated data indicate that approximately 10% to 20% of patients have metastatic disease at the time of surgery in the absence of lymph node involvement [4]. Moreover, it has been reported that up to 30% of patients in whom lymph nodes are not affected experience recurrence within 10 years after surgery [5].

Early hematogenous spread has been proposed as a prognostic indicator that would explain the errors in prognoses established in the initial stages of the disease. Detection of disseminated tumor cells (DTC) in bone marrow is not uncommon, but there are substantial differences (2%-60%) in the reported rates. The incidence seems to be lower and more consistent in the early stages of disease: 13.3% at diagnosis [6], 31% in patients with stage I-II [7], and 19% in T0-2 tumors [8]. The independent prognostic value of DTC remains when it is assessed together with lymph node involvement [9] and in multivariate analysis with clinical parameters of survival in patients with stage I, II, and III disease [8].

Microarray analysis has identified breast cancer subtypes with differing gene expression profiles [10,11]. These subtypes have been correlated with clinical outcome, and the impact of subtype on response to neoadjuvant chemotherapy has been evaluated [12]. Certain easily assessable markers can be used to approximate the breast cancer subtype. Specifically, by determining the estrogen receptor (ER), progesterone receptor (PR), and HER2 status of a tumor, breast subtype can be approximated as follows: luminal A (ER+ or PR+ and HER2-), luminal B (ER+ or PR+ and HER2+), HER2+/ER- (ER- and PR- and HER2+), and triple-negative (ER- and PR- and HER2-) [13,14].

The aim of this study was to determine the incidence and prognostic value of DTC present in bone marrow of patients with early-stage breast cancer, and the relationship of this factor with lymph node involvement assessed by SLN biopsy and other clinical parameters of prognostic interest, including breast cancer subtypes based on ER, PR, and HER2 status.

## Methods

This is a prospective, observational study, in which breast carcinoma patients were evaluated for the presence of DTC in bone marrow. The study was conducted at University Hospital Germans Trias i Pujol in Badalona (Spain). The recruitment period was between 2002 and 2004, and last follow-up was in 2009.

The study population included all consecutive women diagnosed with breast carcinoma in an early stage (tumor size < 3 cm, clinically- and sonographically-negative axillary nodes, and no known metastasis) who were referred for surgical treatment (mastectomy or lumpectomy, and SLN biopsy) during the recruitment period. The exclusion criteria were a history of surgery or axillary radiation therapy, neoadjuvant chemotherapy, pregnancy or lactation, and patients with clinical or personal characteristics that would hinder monitoring. Patients signed an informed consent form for participation in the study. The study was approved by the Ethics and Clinical Research Committee of our hospital and was designed in compliance with the Helsinki Declaration.

### Sentinel Node Detection and Biopsy

**Figure 1:**
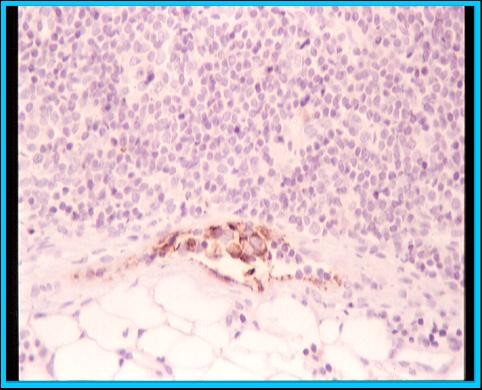
**Sentinel node micrometastasis**.

Lymphoscintigraphy was performed 2 hours after intratumor administration of 2 mCi (74 MBq) of 99mTc colloidal albumin. Tracer administration was guided by sonography or mammography; hence, the radioguided occult lesion localization (ROLL) technique was also available. After intraoperative SLN detection and biopsy, the specimen was evaluated for the presence of tumor cells both intraoperatively with a fast variation of May Grünwald-Giemsa staining, and definitively by multilevel sectioning and hematoxylin-eosin staining. When tests were negative, immunocytochemistry using an anticytokeratin antibody (CAM 5.2) was performed (Figure 1).

### Immunocytochemical Determination of Disseminated Tumor Cells in Bone Marrow

The method used for the immunocytochemical determinations was based on the standards set by the European group for standardization of tumor cell detection of the International Society of Hematotherapy and Graft Engineering (ISHAGE) [15], with an adaptation to our laboratory.

**Figure 2:**
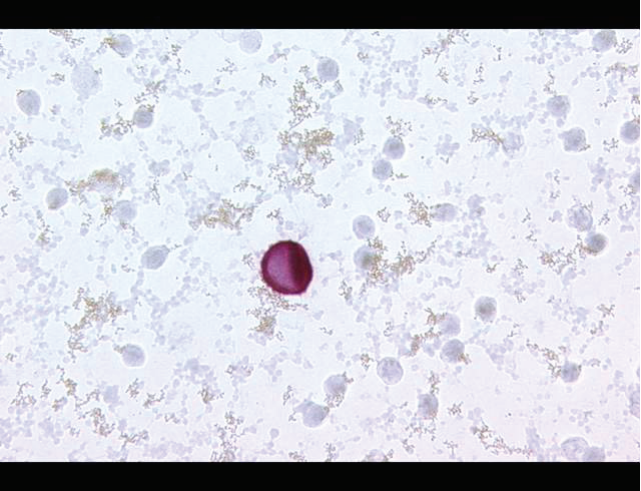
**Isolated tumor cell in bone marrow**.

Prior to the surgical procedure and while the patient was anesthetized, bone marrow aspirates were collected from both anterior iliac crests to obtain a 10-mL specimen. The core sample was immediately processed by density centrifugation using Lymphoprep (Nycomed, Oslo, Norway). Centrifugation results in redistribution of the components according to density, so that mononuclear cells and breast tumor cells are presented in an easily separated layer. Cells were recovered, counted, and prepared for immunocytochemical staining. Marrow aspirate samples were analyzed quantitatively using the alkaline phosphatase-alkaline antiphosphatase technique, seeking epithelial cells with cytokeratin expression. The monoclonal antibody, antiCK A45-B/B3 (Micromet, Munich, Germany), which is a combination of antibodies directed against cytokeratin 8, 18, and 19, was used in a total of 2 × 10^6 ^cells (Figure 2).

Before applying the technique in clinical practice, it was used to evaluate bone marrow from 10 patients without cancer to validate its specificity. In addition, the technique was calibrated using MCF-7 breast cancer cells at different dilutions spiked into mononuclear cells from patients without breast cancer.

During the clinical phase, all breast cancer specimens were systematically examined in parallel with controls consisting of slides stained with isotype-matched immunoglobulin. Morphological analysis with light microscopy was used in all specimens to rule out nonspecific staining in nonmetastatic cells. Bone marrow specimens and sentinel nodes were examined by independent observers.

### Patient Follow-Up

Follow-up was carried out according to the standard hospital protocol established by the breast pathology unit. All patients received adjuvant chemotherapy and/or hormonal therapy according the institutional and international guidelines [16], starting no later than 6 weeks after surgery. In addition, all patients treated with conservative breast surgery received external radiotherapy. Subsequently, clinical follow-up visits were scheduled every 3 months during the first 2 years and every 6 months up to 5 years. These visits included a physical examination, in which particular attention was placed on lymph node evaluation and identification of metastasis. Tumor markers were assessed every 6 months. Chest radiography and mammography were performed once a year. Bone scintigraphy, abdominal ultrasound study, or computed tomography (CT) scanning was done in cases of suspected relapse.

### Study Variables and Data Analysis

The study variables were patient age, tumor-related characteristics including size, location, radiological presentation, grade, lymphovascular invasion and histological type, and hormone receptor (HR) status, including estrogen, progesterone, and HER-2 overexpression. Based on ER, PR, and HER2 status, tumors were classified into three subtypes: ERBB2+ (HR - and Erb-B2 receptor +), triple negative (HR and HER-2-negative) and luminal (combination of positive estrogen receptor with any progesterone result or Erb-B2 status. SLN surgical and biopsy results, and DTC findings from bone marrow aspirates were also recorded. The follow-up variables included type and duration of treatment, follow-up period, and overall and disease-free survival.

A descriptive analysis was performed of all variables. Qualitative variables were described using frequency tables for the different categories, and quantitative variables as the mean and standard deviation (SD).

Associations between variables of interest were analyzed. The chi-square test was used to compare qualitative variables. Comparisons between quantitative and qualitative variables were performed with the Student *t *test (dichotomous variables) or ANOVA (variables with more than two categories), taking into account the possibility of using nonparametric tests.

For hypothesis testing, a two-tailed *P *value of 0.05 and power of 80% were used. The data analysis was performed with SPSS for Windows (version 15.0).

## Results

A total of 104 patients with mean age of 55.6 years (30-87) were studied. Mean tumor size was 1.87 cm (range, 0.5-4 cm).

Lymphoscintigraphy was performed 2 to 18 hours after tracer injection. There was no tracer migration to the axillary region in 6 cases, making subsequent lymphadenectomy necessary. One node was detected in 59 patients, 2 nodes in 31 patients, 3 nodes in 7 patients, and 4 nodes in 1 patient. Internal mammary drainage occurred in 20 patients.

Axillary SLN biopsy was positive in 27 of 98 cases (28%) (Table 1). Micrometastasis was found in 17 patients (63%), but 4 of them did not undergo lymphadenectomy because they entered in the AATRM trial, a multicenter clinical trial conducted in our center [17]. A second node was found to be affected in 4 of the 13 lymphadenectomies carried out (31%), in one case with micrometastasis. Of the 10 patients who underwent lymphadenectomy after detection of SLN macrometastasis, nodes other than the SLN were positive in 4 patients. There were no cases of internal mammary SLN involvement. Lastly, in 2 of the 6 patients with no tracer migration, lymphadenectomies were positive, yielding a total of 29 patients with lymph node involvement.

**Table 1:** Results of sentinel lymph node biopsy

	Patients	Positive SN	Lymphadenectomy	Positive node other than SN
No axillary migration	6		6	2

Axillary drainage	98	17 micromets	13	4
		10 macromets	10	4

Internal mammary drainage	20	0		

Abbreviations: macromets, macrometastasis; micromets, micrometastasis; SN, sentinel node

Iliac crest bone marrow aspirates were successfully collected in all patients, with bilateral extraction in 85% of them. The mean volume obtained was 10 mL (range, 4-15 mL). One or two isolated tumor cells were found in 23 patients (22%).

Of the 23 patients with positive bone marrow aspirates, lymph node involvement was negative in 18 patients and was positive in 5 patients, 1 of whom showed micrometastasis. Thus, only 5 patients (4.8%) tested positive to both lymph node and bone marrow involvement, with no overall correlation between the two routes of metastatic spread (chi-square = 0.232; *P *= 0.63) (Table 2).

**Table 2:** Results for lymph node and bone marrow involvement

	BMi+	BMi-	
LNi+	5 (4.8%)	24 (23%)	

LNi-	18 (17.3%)	57 (54.8%)	ns

Abbreviations: LNi, lymph node involvement; BMi, bone marrow involvement; ns, nonsignificant

The results of the evaluation of clinical and pathological characteristics are shown in Table 3. Lymph node spread was related to younger age (*P *< 0.05), whereas larger tumor size, infiltrating ductal histological pattern, high grade, and lymphovascular invasion were relevant, but non-significant, factors. Hematogenous spread was related to older age (*P *< 0.05), radiologic presentation as a nodule (*P *< 0.05), and internal/central mammary localization (*P *< 0.05), whereas high grade, and positive estrogen and progestogen receptor status were present as relevant, but not significantly related.

**Table 3:** Comparative analysis of clinical characteristics and prognostic factors in patients with lymph node or bone marrow involvement

	LNi- (n = 75)	LNi+ (n = 29)		BMi- (n = 81)	BMi+ (n = 23)	
Age, y	57.4	50.8	*P *= 0.003	53.6	62.4	p = 0.007

Tumor size, cm	1.88	2.16	ns	1.97	1.88	ns

Location						
External quadrants	68%	65%	ns	73%	48%	
Internal quadrants	32%	35%		27%	52%	p = 0.045

Radiological presentation						
Nodule	71%	76%	ns	67%	91%	
Microcalcification or distortion	29%	24%		33%	9%	p = 0.04

Histological type						
Ductal infiltration	85%	90%	ns	87%	87%	
No ductal infiltration	15%	10%		13%	13%	ns

Tumor grade						
I	27%	12%	ns	23%	22%	
II-III	73%	88%		77%	78%	ns

Lymphovascular invasion	11%	21%	ns	13%	13%	ns

Positive estrogen receptor status	70%	70%	ns	69%	76%	ns

Positive progesterone receptor status	59%	63%	ns	58%	67%	ns

Positive Erb-B2 receptor status	33%	19%	ns	28%	30%	ns

Abbreviations: LNi, lymph node involvement; BMi, bone marrow involvement; ns, nonsignificant

According to the established hospital protocols, patients received adjuvant chemotherapy (71%) and hormonal therapy (29%); within this total, 12% were given a sequential combination of both treatments. Considering that patients were included before the publication of the results of adjuvant trastuzumab, none of the patients included received this drug as adjuvant therapy [18-20]. Patients who underwent conservative surgical treatment (80%) received radiotherapy. Follow-up reached 60 months in all patients (maximum 90 months). The patients' clinical course and the relationship between clinical course and lymphatic and hematogenous involvement are shown in Table 4. Cancer-related deaths occurred in 4 patients, and 4 others presented disease relapse, consisting of local progression (2 patients) or distant disease recurrence (2 patients). None of these patients were among those included in the AATRM trial. Neither lymphatic involvement (RR = 1.3; 95% CI = 0.12-13.8) nor hematogenous involvement (RR = 1.8; 95% CI = 0.17-18.9) reached a significant overall risk.

**Table 4:** Clinical outcome

	LNi-	LNi+		BMi-	BMi+	
Time, months	56.5	55.5	ns	55.9	56.9	ns

Disease free						
no	6 (6%)	2 (2%)		5 (5%)	3 (3%)	

yes	69 (66%)	27 (26%)	ns	76 (73%)	20 (19%)	ns

Overall survival						
died	3 (3%)	1 (1%)		2 (2%)	2 (2%)	

alive	72 (69%)	28 (27%)	ns	79 (76%)	21 (20%)	ns

Abbreviations: BMi, bone marrow involvement; LNi, lymph node involvement; ns, nonsignificant

Analysis of the relationships between the different breast cancer subtypes and disease-free survival showed a higher incidence of HER-2-positive tumors and HR-negative plus HER-2-negative tumors in patients who relapsed or died due to the disease (*p *< 0.01); whereas no relationship was found between these subtypes and the presence of DTC in bone marrow (Table 5).

**Table 5:** Correlation of clinical outcome with breast subtypes

	ERBB2+	Triple-negative	Luminal	
DTC in bone marrow				
no	5 (71%)	15 (79%)	61 (78%)	ns
yes	2 (29%)	5 (21%)	4 (22%)	ns

Disease-free				
no	2 (29%)	4 (21%)	2 (3%)	p = 0.001
yes	5 (71%)	15 (79%)	76 (97%)	

Abbreviations: DTC, disseminated tumor cells; ns, nonsignificant

## Discussion

The main objective of the present study was to estimate the incidence of disseminated tumor cells in bone marrow as a criterion for assessing hematogenous metastatic spread in patients with breast carcinoma. In addition, the correlations between this feature and axillary lymph node involvement were determined in patients who underwent sentinel node biopsy. The study, which was conducted between 2002 and 2009, assessed disease-free and overall survival.

The immunocytochemistry results obtained in bone marrow using a combination of antibodies directed against cytokeratins 8, 18, and 19, indicate a 22% incidence of DTC, which is in the reported range (12.5%-28%) documented in the early stages of breast carcinoma[21,22].

Although a higher frequency of DTC was found in the subgroup of patients who experienced a relapse (13% vs. 6%, ns) or died of cancer (9% vs. 3%, ns), the statistical study did not validate the prognostic value of the results. This is likely because a low rate of events (8 of 104 patients) occurred over the follow-up period, as would be expected in patients surgically treated at an early stage of the disease and receiving systemic chemotherapy or hormone therapy, as well as additional radiotherapy in those with conservative surgery.

Other authors have reported results that are more decisive. In a review published in 2005 by Braun and Vogl [23], which included a large pool of patients in different disease stages, the presence of DTC in bone marrow in the low-risk subgroup (patients who had tumors no larger than 2 cm and no lymph node involvement, and who were not undergoing adjuvant therapy) was associated with a 3.65-fold increase in the risk of breast cancer mortality and a 2-fold increase in the risk of distant metastasis during the first 5 years. In this study and others [24] DTC is described to have independent prognostic value, among other known prognostic factors. Nonetheless, other authors consider that the long-term predictive capacity of this factor is not conclusive in patients with initial-stage disease [8].

The differences in the results of these studies may be explained by the differing methods and study designs used, small size of the study groups, heterogeneity of the patients included, short follow-up periods, and differing treatment regimens applied. In addition, bias can occur when heterogeneous pools of patients from several institutions are investigated or when results from patients in early disease stages are combined with those from patients with known metastatic disease [25-29]. This is not the case of our study, which focused on a homogeneous group of patients who had no evidence of disseminated disease at the time of primary surgery, were receiving adjuvant systemic treatment, and were evaluated in terms of survival for a maximum of 6 years of follow-up.

Another aspect is the diversity of techniques used by different groups for determining bone marrow dissemination. We used an immunoassay that meets the ISHAGE committee's recommendations for detecting tumor cells in bone marrow [15] to ensure high reproducibility of results. To determine the detection capability, we tested the sensitivity of the method with isolated MCF-7 mononuclear cells, which show high cytokeratin expression. Based on the results, we did not contemplate the use of cell enrichment methods with magnetic particles [30]. Furthermore, we did not consider it appropriate to perform peripheral blood determinations because they require a larger sample volume and offer less reproducible results [31,32]. These issues have been extensively discussed in the meta-analysis of Weinschenker et al [33], which includes the results from 9 series published between 1991 and 2002. The findings are discussed in light of the heterogeneity of the methods used in a heterogeneous pool of patients treated with different regimens.

Lymph node involvement investigated in patients who underwent SLN biopsy or complete axillary lymph node dissection yielded 28% of positive results (29/104), which is within the expected number for patients with early disease. In addition, the 63% incidence (17/27) of lymph node microinvasion could be expected, considering that these patients had undergone ultrasound study and clinical examination, which are highly effective for ruling out macroscopic nodal involvement. It may be surprising that none of the biopsies of the internal mammary area were found to be positive, even taking into account the high percentage of locations in the inner quadrants and central area (around 30%).

Simultaneous bone marrow and lymph node involvement was detected in 5 of 104 patients, in 1 case by micrometastasis. These findings suggest that tumor cell dissemination to the two sites rarely occurs simultaneously in the early stages of the disease, as has been reported by other authors. In the series of Braun et al [27], only 1% (2/150) of patients simultaneously presented micrometastasis via lymphatic and hematogenous route, and Gerber et al [34] found only 5% of micrometastasis in the two specimens. More recently, Langer et al [35] described similar rates of nodal involvement and BM micrometastasis, but agreement was 20% between the two specimens.

According to the recently reported results of the ACOSOG Z-010 study [36] in a series of 5539 patients, DTC in bone marrow was a significant predictor of an increased risk of death, whereas SLN micrometastasis was not a significant prognostic factor in this regard. However, BM metastasis was detected in 105 of the 3491 patients examined (3%). In our experience, DTC in bone marrow was also associated with a higher risk of relapse and death, but differences were not statistically significant (see Table 4).

As occurred in other studies [17,24], hematogenous involvement was linked to other parameters of prognostic interest, such as older age (*P *< 0.05), radiologic presentation as a nodule (*P *< 0.05), and location in internal quadrants or central (*P *< 0.05).

Lastly, the relationship between the events occurring during follow-up and the different breast cancer subtypes of the tumor was examined. Not surprisingly, patients who experienced a relapse showed a significant relationship with the HR-negative plus HER2-2-negative tumors, and with HER-2-positive tumors (*P *< 0.001), which are known to be associated with poorer survival [18-20]. As was shown by our results, this fact does not seem to be related with a higher incidence of DTC in these subtypes. Considering that patients were included in the study before the publication of the studies using adjuvant trastuzumab, patients with HER-2-positive tumors included did not receive this adjuvant treatment; this fact could explain the poorer prognosis of this subgroup.

## Conclusions

To conclude, determination of tumor cells by immunocytochemistry in bone marrow aspirates is a valid parameter for assessing early hematogenous spread in patients with initial-stage breast carcinoma; this finding was documented in 22% of patients. Hematogenous spread, which was seen in 28% of patients as an independent feature, does not seem to occur in parallel to lymph node invasion. The survival study showed a higher frequency of disease-related events in patients presenting with hematogenous involvement. Nonetheless, the biological profile of the tumor showed higher power for identifying the subgroup of patients with poorer survival.

Establishment of comparable research protocols in both methodological and analytical aspects, as well as in selection of patients, is needed to derive conclusions regarding the value of these complex determinations. The classic prognostic parameters remain highly important and a simpler one, the biological profile, is emerging as an easily assessable descriptor with considerable clinical implications.

## List of abbreviations

SLN: Sentinel lymph node; DTC: Disseminated tumor cells; ROLL: Radioguided occult lesion localization; ISHAGE: International Society of Hematotherapy and Graft Engineering; HR: Hormone receptor

## Competing interests

The authors declare that they have no competing interests.

## Authors' contributions

MS designed the study, carried out the immunocytochemical determinations of bone marrow specimens, and coordinated the study, which corresponds to a substantial part of her doctoral thesis (Autonomous University of Barcelona). MM participated in patient monitoring. EC carried out the sentinel node histochemical analyses. JJ, MR and JG are surgeons from the breast units of their respective hospitals. AM participated in patient diagnosis and monitoring. AB and MF conceived of the study and participated in its design.

All authors read and approved the final manuscript.

## Pre-publication history

The pre-publication history for this paper can be accessed here:

http://www.biomedcentral.com/1471-2407/11/252/prepub
